# Comparison between invasive and non-invasive assessment of blood pressure in hypertensive disorders of pregnancy

**DOI:** 10.1186/s43044-021-00172-7

**Published:** 2021-05-25

**Authors:** Ayman Khairy M. Hassan, Ayman H. Shaamash, Asmaa G. Mohamed, Salwa R. Demitry, Nady A. Razik

**Affiliations:** 1 Assiut University, Assiut, Egypt; 2 Faculty of Medicine, Assiut University, Assiut, Egypt; 3 Cardiology Department, Faculty of Medicine, Assiut University, Assiut, Egypt

**Keywords:** Hypertensive disorders of pregnancy, Invasive BP, Oscillometric non-invasive BP, Mobil-O-Graph

## Abstract

**Background:**

The management of hypertensive disorders of pregnancy (HDP) during hospitalization requires an accurate blood pressure (BP) measurement, mainly by invasive intra-arterial reading. Nevertheless, little is known about the precision of non-invasive (NI) central BP measurements in HDP. We aimed to assess the accuracy of NI central BP assessment in comparison to invasive BP measurement in HDP.

This cross-sectional study included all patients with HDP that were admitted to university hospitals for high BP control, from December 2018 till December 2019, and 10 healthy matched non-hypertensive controls. Patients were compared for demographic, anthropometric, and echocardiographic data. In all subjects, invasive BP assessment was done by radial arterial cannulation and NI assessment of BP was performed by an oscillometric automated device (Mobil-O-Graph); the comparison was done after initial control of BP.

**Results:**

One hundred patients were included and divided into 3 groups (pre-existing hypertension (HTN), gestational HTN, and pre-eclampsia). There was no statistically significant difference between NI central and invasive methods in measuring both systolic BP (SBP) (126.39 ± 14.5 vs 127.43 ± 15.3, *p* = 0.5) and diastolic BP (82.41 ± 9.0 vs 83.78 ± 8.9, *p* = 0.14) among the total studied population. A strong positive correlation was found between NI central and invasive SBP (*r* = 0.96, *p* < 0.001). HDP was associated with an increase in arterial stiffness, left ventricular diastolic dysfunction, and complications.

**Conclusion:**

Non-invasive measurement of BP using oscillometric automated devices is as accurate as the invasive method, and it is a practical safe method in pregnant women with hypertensive disorders (CTR no. = NCT04303871).

## Background

Hypertensive disorders of pregnancy (HDP), an umbrella that includes pre-existing and gestational hypertension, pre-eclampsia, and eclampsia, complicate up to 10% of pregnancies and represent a significant cause of maternal and perinatal morbidity and mortality [1].

The definition of hypertension (HTN) in pregnancy is based only on office (or in-hospital) BP values [systolic BP (SBP) ≥ 140 mmHg and/or diastolic BP (DBP) ≥ 90 mmHg]. However, the pathophysiology of HTN in pregnancy leading to a significant difference between central and peripheral hemodynamics all through pregnancy, thus necessitating the use of other methods to follow up HDP [2, 3]. Brachial BP may differ from central BP (systolic BP measured at the level of the ascending aorta), which is more important because of its predictive value for cardiovascular events in all patients including pregnant women [4]. As central BP was one of the explanations of the increased stroke risk in atenolol arm in landmark LIFE trial [5].

Invasive blood pressure measurement via arterial cannulation, commonly from a major artery, is considered to be the most accurate method of blood pressure measurement in those patients that need intensive care admission for control of high BP during pregnancy [6]. However, arterial cannulation is associated with increased risks, such as hematoma, thrombosis, and infection; thus, alternative non-invasive blood pressure measurements may be used [7]. Nevertheless, little is known about the accuracy of invasive and non-invasive methods for central BP measurements in hypertensive pregnant women.

In this study on pregnant women with different types of HTN, we studied the accuracy of non-invasive central blood pressure assessment by an oscillometric automated device (Mobil-O-Graph) in comparison to the standard invasive blood pressure measurement.

## Methods

### Study design and setting

This cross-sectional study was conducted between the 1st of December 2018 to the 1st of December 2019 at a university hospital. The study was approved by our university institutional review board (RD no. = 17101032) and complied with the Declaration of Helsinki. Written informed consent was obtained from all patients (clinical trial registration no. = NCT04303871).

### Study participants

We included all pregnant women admitted to our university women’s health hospital during this period and diagnosed to have HDP that necessitate hospital management by anti-hypertensive medications. Exclusion criteria were patients with a history of cardiac diseases, chronic kidney disease, endocrine diseases such as hyperthyroidism, and conditions that prevent arterial cannulation as severe bleeding disorders or peripheral arterial disease. One hundred seventeen patients were recruited; 17 patients were excluded (10 patients refused to join the study due to fear of intra-arterial cannulation and 7 patients showed a failure of the cannulation technique). Another group of 10 healthy non-hypertensive non-pregnant women, with matched age, weight, and height, were also included in the study as a control group to validate BP measurements’ accuracy, both invasive and central non-invasive techniques. We divided our patients into 3 groups according to recent 2018 ESC guidelines of HTN [8] (pre-existing hypertension, gestational hypertension, and pre-eclampsia group).

### Study variables and data measurements

All patients were subjected to a full medical and an obstetric history taking and clinical examination, including BP measurement by 3 different methods and echocardiographic examination. Exclusion of albuminuria was done at the time of hospital admission as routine work to all participants using the dipstick method owing to its sensitivity, convenience, and being widely available.

#### Blood pressure assessment

In the blood pressure assessment in all studied groups, BP measurement was done after control of initial blood pressure on the 3rd or 4th day of hospital admission by 3 different methods including:

A) Office blood pressure measurement using sphygmomanometer: According to ESC guidelines of hypertension 2018, BP was measured in the sitting position (or the left lateral recumbent) with an appropriately sized arm cuff at heart level and using Korotkoff V for DBP, and an average of 3 readings was done [8]
B) Non-invasive central BP monitoring in a quiet, temperature-controlled examination room: Three measurements with a 2-min break between them were taken to all patients in a sitting position with an adequately sized cuff. The Mobil-O-Graph 24 h NG (IEM, Stolberg, Germany) with inbuilt ARC Solver (Austrian Institute of Technology, Vienna, Austria) and its blood pressure detection unit is validated according to the British Hypertension Society and European Society of Hypertension recommendations [9, 10]. Algorithms were used to obtain central blood pressure readings: brachial systolic and diastolic pressures. Pulse wave velocity (PWV) values in relation to age, used for the detection of the degree of arterial thickness related to hypertension and augmentation index (AIx) of the central pressure, were recorded. AIx was considered normal if less than − 10% [11]
C) Invasive blood pressure measurement through percutaneous radial artery cannulation by 20-gauge Teflon cannula under aseptic conditions after palpation of the artery as a guide. Cannulation was done by a single experienced operator. The arterial cannula was connected to a disposable tubing system and flushed frequently with a heparinized 0.9% saline solution. The tubing liquid was connected to a transducer (Auto Transducer®; ACE Medical, Inc., Goyang, Korea). The transducer was kept horizontally at the level of the patient’s 4th intercostal anterior axillary line, and the radial access was at the same height of the cuff. We perform zeroing by opening the transducer to the atmospheric pressure and electronically zeroing the system. We recorded the arterial blood pressure signals by using a bedside monitor (GE Datex-Ohmeda S/5TM Anesthesia Monitor, Helsinki, Finland).

The BP measurements using the three mentioned methods were conducted by 2 different clinicians (A.G and M.K) in 2 different occasions using the standardized measuring procedures and reported their reading separately to the data collecting hub nurse. The inter-observer agreement was calculated with weighted Kappa statistics and showed good agreement (k = 0.95, *P* = 0.001).

#### Resting transthoracic 2D echocardiography (ECHO)

Resting transthoracic 2D echocardiography (ECHO) was performed on all patients before discharge from the hospital. ECHO was performed in the left lateral decubitus position using VIVID S5 instrument, GE Medical Systems, Horten, Norway, based on the recommendations of the European Association of Echocardiography (EAE) and American Society of Echocardiography (ASE) [12]. Assessment of LV end-systolic, end-diastolic dimensions, and M-mode ejection fraction (EF) is from parasternal short-axis view. The apical 4- and 2-chamber views were acquired for calculation of Simpson’s LV volumes and EF (Simpson’s EDV, ESV, and EF). We assessed LV diastolic function according to the EAE and ASE guidelines, through the calculation of trans-mitral E max, A max, E/A ratio, E/e′ ratio, and left atrial volume index. E/e′ was calculated as the average ratio between septal E/e′ and lateral E/e′. Early (septal e′ and lateral e′) and late (septal a′ and lateral a′) velocities of septal and lateral mitral annulus, and their average e′/a′ ratio was calculated. Evaluation of left ventricular hypertrophy (LVH) was done as well.

### Statistical analysis

We presented the categorical variables as counts and percentages then compared by Pearson chi-square analysis or Fisher’s exact test. We tested the normal distribution of our continuous data by Kolmogorov-Smirnov test. We presented continuous and normally distributed data as mean ± SD and were compared by unpaired *t* test. Analysis of variance (ANOVA) test was used to compare differences between more than two groups. The inter-observer agreement was calculated with weighted Kappa statistics. Correlations were done by Spearman correlation coefficient test. All *P* values are two-tailed, and statistical significance was defined if *P* < 0.05. Our analyses were performed with SPSS version 22.0 statistical software (SPSS Inc., Chicago, IL, USA).

## Results

The current study examined 117 patients for eligibility. Seventeen patients were excluded (10 patients refused to join the study due to fear of intra-arterial cannulation and 7 patients showed a failure of the cannulation technique). Finally, the study included 100 pregnant participants and 10 healthy controls (non-hypertensive and non-pregnant women) who were seen and examined at a Women Health Hospital and a University Heart Hospital. We divided our patients into 3 main groups, defined according to 2018 ESC guidelines of hypertension [8] into pre-existing hypertension represented 7 (7%) cases, gestational hypertension represented 24 (24%) cases, and pre-eclampsia group represented 69 (69%).

### Demographic and clinical data of the study population

The mean age of the study population was 29.16 ± 6.6 years with no significant difference between all 3 groups. The lowest number of previous pregnancies was seen in the pre-eclampsia group (2 ± 2) as shown in Table 1. A group of 10 healthy non-hypertensive women with matched age, weight, and height was used as a control group. All the study patients were on regular oral anti-hypertensive medications (nifedipine, labetalol, alpha-methyl Dopa, or a combination). We found that the largest percentage of the three groups was on combination therapy.

**Table 1:** Demographic, clinical and echocardiographic data of the study groups

	Total patients (***n*** = 100)	Pre-existing hypertension (***n*** = 7)	Gestational hypertension (***n*** = 24)	Pre-eclampsia (***n*** = 69)	***P*** value
Age (years)	**29.16 ± 6.6**	**26.25 ± 6.8**	**29.92 ± 6.8**	**29.24 ± 6.5**	**0.4**
Weight (kg)	**88.84 ± 12.3**	**89.13 ± 13.1**	**91.38 ± 10.8**	**87.91± 12.8**	**0.5**
Height (cm)	**161.04 ± 4.9**	**159.38 ± 3.3**	**162.83 ± 3.8**	**160.6 ± 5.2**	**0.09**
Arm circumference (cm)	**27.5 ± 4.9**	**28.5 ± 3.2**	**27.5 ± 4.9**	**27.4 ± 5.1**	**0.3**
Body surface area (m^2^)	**1.92 ± 0.15**	**1.92 ± 0.17**	**1.98 ± 0.14**	**1.98 ± 0.16**	**0.3**
Body mass index (kg/m^2^)	**34.05 ± 4.5**	**34.41 ± 3.9**	**35.57 ± 5.9**	**33.77 ± 4.1**	**0.2**
Number of previous pregnancies	**2 ± 2**	**3 ± 2**	**3 ± 1**	**2 ± 2**	0.006*
Gestational age (weeks)	**32.56 ± 4**	**33.38± 2.4**	**33.25 ± 3.5**	**32.22 ± 2.4**	**0.5**
Diabetes mellitus	**18 (18%)**	**2 (25%)**	**6 (25%)**	**10 (15%)**	**0.4**
History of hypertension during previous pregnancies.	**32 (32%)**	**3 (38%)**	**7 (29%)**	**22 (32%)**	**0.9**
Albuminuria	**69 (69%)**	**0**	**0**	**69 (100%)**	0.01*
**Medications used**					0.01*
Alpha methyl dopa	35 (35%)	3 (42.8%)	6 (25%)	26 (37.6%)	
Labetalol	3 (3%)	0	1 (4.1%)	2 (2.8%)	
Nifedipine	25 (25%)	2 (28.5%)	7 (29.1%)	16 (23.1%)	
Combination	37 (37%)	2 (28.5%)	9 (37.5%)	26 (37.6%)	
**Echocardiography**					
**LA volume index (ml/m**^**2**^**)**	34.22 ± 1.7	33.38 ± 1.6	34.63 ± 1.9	34.65 ± 1.6	0.15
**LV mass index**	63.65 ± 12.1	66.38 ± 7.5	59.83 ± 12.4	64.74 ± 12.4	0.19
**Biplane Simpson’s EF %**	65.15 ± 3.9	65.38 ± 3.8	65.29 ± 4	64.79 ± 3.8	0.8
**E max (m/s)**	0.83 ± 0.24	1.07 ± 0.23	0.84± 0.25	0.80 ± 0.23	0.01*
**E/A ratio**	0.99 ± 0.35	1.26 ± 0.26	1.04 ± 0.37	0.93 ± 0.33	0.02*
**e′ lateral (m/s)**	0.10 ± 0.026	0.12 ± 0.02	0.10 ± 0.02	0.09 ± 0.02	0.01*
**e′ septal (m/s)**	0.077 ± 0.05	0.10 ± 0.02	0.08 ± 0.03	0.07 ± 0.02	0.003*

Data was expressed in the form of mean ± SD, frequency (percentage). *P* value was significant if < 0.05. *n* the number of patients, *cm* centimeters, *kg* kilograms, *m*^*2*^ square meters, *EF* ejection fraction, *LA* left atrium, *LV* left ventricle. * Significant values are labelled

### Different BP measurements

#### Non-invasive central BP vs invasive BP

**Fig. 1:**
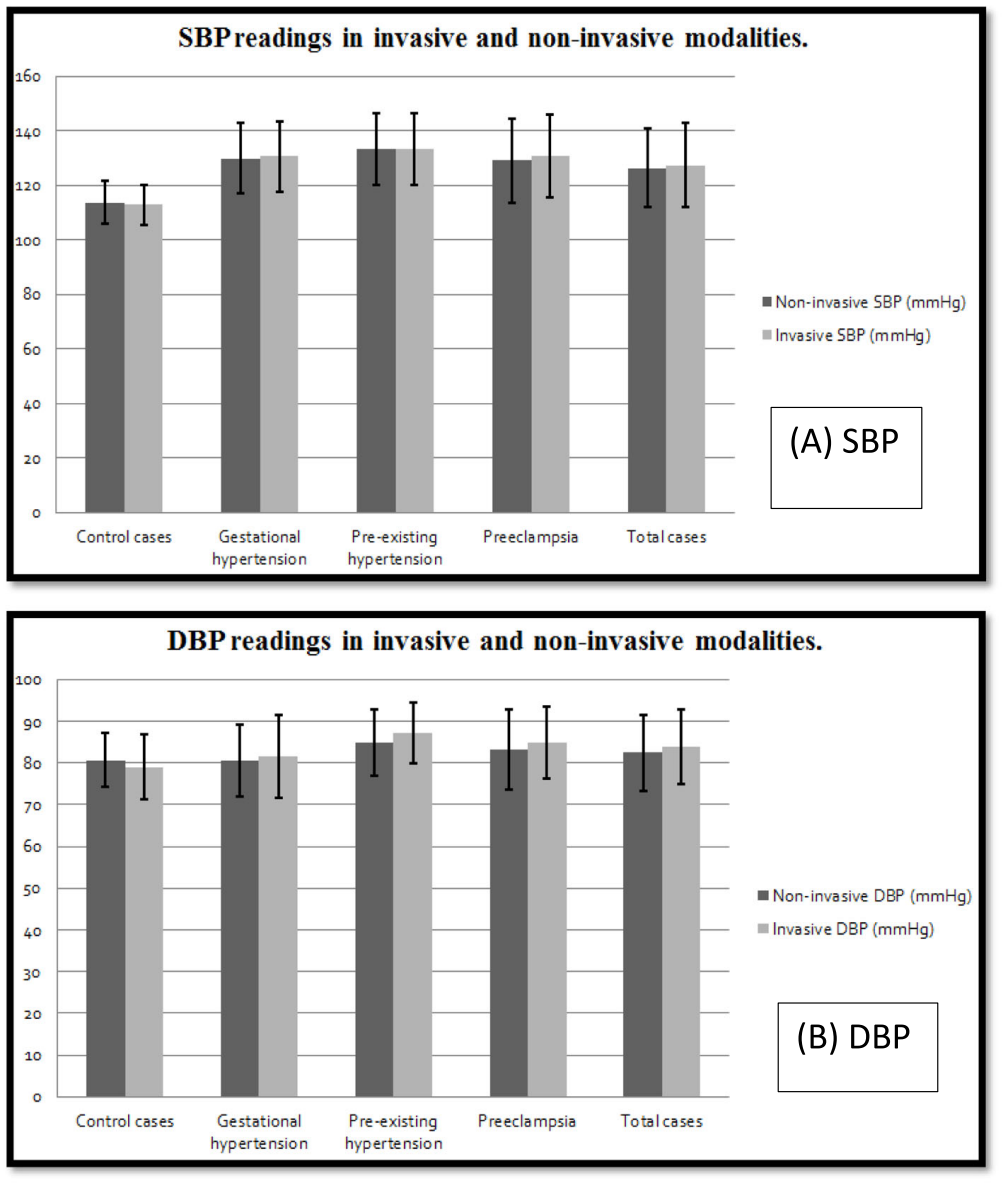
**a** Mean systolic blood pressure in invasive vs central non-invasive modality. **b** Mean diastolic BP in invasive vs central non-invasive modality. SBP, systolic blood pressure; DBP, diastolic blood pressure

Results showed no significant difference between non-invasive central and invasive methods in measuring both SBP (126.39 ± 14.5 vs 127.43 ± 15.3, *p* = 0.5) and DBP (82.41 ± 9.0 vs 83.78 ± 8.9, *p* = 0.14) among the total studied population. The same non-significant difference was noted among the studied groups, including the control, as shown in Table 2 and Fig. 1.

**Table 2:** Accuracy of non-invasive central against invasive SBP and DBP among the studied cohort

	SBP		***P*** value	DBP		***P*** value
	Invasive (mmHg)	Noninvasive central (mmHg)		Invasive (mmHg)	Noninvasive central (mmHg)	
**Control (*n*** **= 10)**	**112.90 ± 7.2**	**113.70 ± 7.84**	**0.2**	**79.00 ± 7.8**	**80.70 ± 6.5**	**0.098**
**Gestational hypertension (*n*** **= 24)**	**130.60 ± 13.05**	**129.95 ± 12.9**	**0.07**	**81.54 ± 9.9**	**80.42 ± 8.6**	**0.370**
**Pre-existing hypertension (*n*** **= 7)**	**133.37 ± 13.1**	**133.25 ± 13.2**	**0.5**	**87.14 ± 7.2**	**84.86 ± 7.9**	**0.075**
**Preeclampsia (*n*** **= 69)**	**130.62 ± 15.15**	**129.27 ± 15.45**	**0.06**	**84.91 ± 8.5**	**83.10 ± 9.6**	**0.289**
**Total (*n*** **= 110)**	**127.43 ± 15.3**	**126.39 ± 14.5**	**0.5**	**83.78 ± 8.9**	**82.41 ± 9.0**	**0.141**

Data were expressed in the form of mean ± SD. *P* value was significant if < 0.05. *n* the number of patients, *DBP* diastolic blood pressure, *SBP* systolic blood pressure

**Fig. 2:**
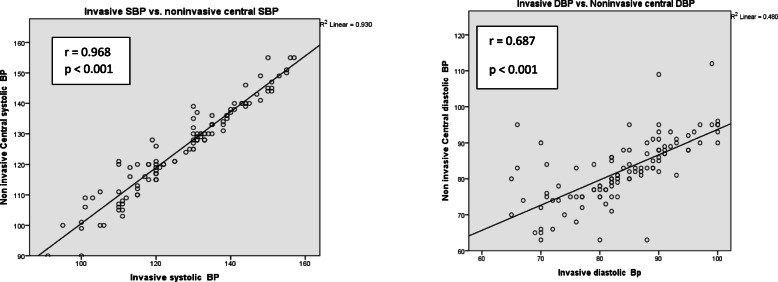
Correlation between invasive (SBP/DBP) and noninvasive central (SBP/DBP) for the total patient population. BP, blood pressure; SBP, systolic blood pressure; DBP, diastolic blood pressure

Using Spearman correlation test, a strong positive correlation was found between noninvasive central SBP and invasive SBP (*r* = 0.968, *p* = < 0.001). Moreover, noninvasive central DBP and invasive DBP showed moderate positive correlation (*r* = 0.687, *p* < 0.001) as shown in Fig. 2.

#### Non-invasive central BP vs sphygmomanometer BP

**Fig. 3:**
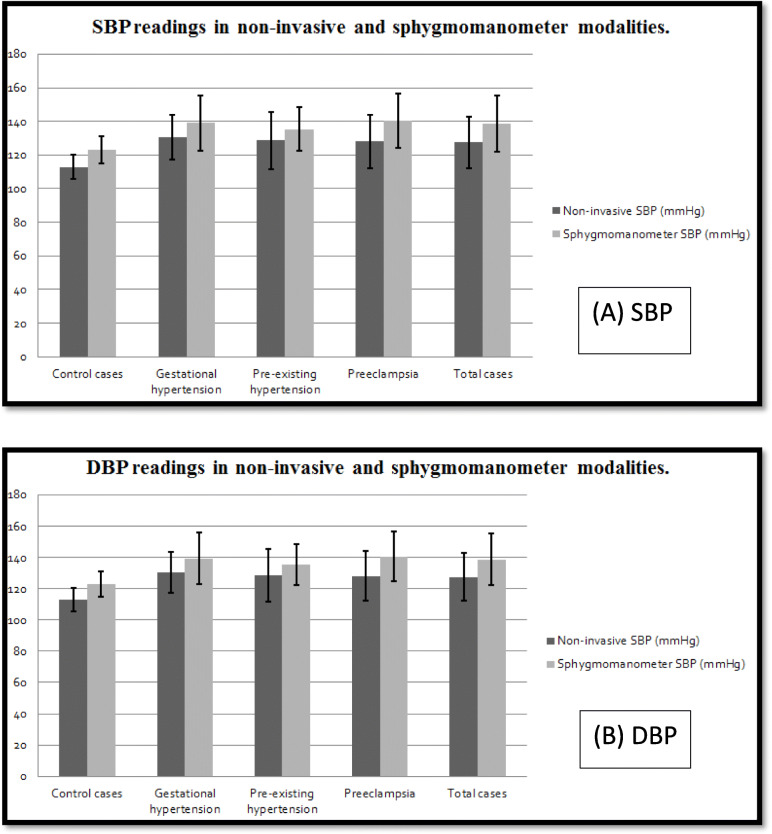
Difference between central non-invasive BP and sphygmomanometer in both. **a** Mean Systolic blood pressure in non-invasive central vs sphygmomanometer modality. **b** Mean diastolic BP in noninvasive central vs sphygmomanometer modality. SBP, systolic blood pressure; DBP, diastolic blood pressure

Table 3 represents the results of comparing non-invasive BP readings to the traditional sphygmomanometer BP measurement. It showed a significant difference between both techniques in measuring SBP among each of the studied groups, including the control, as shown in Fig. 3. SBP readings were significantly lower with the non-invasive central BP method compared to the usual sphygmomanometer method.

**Table 3:** Accuracy of peripheral sphygmomanometer against noninvasive SBP among the studied cohort

	SBP		***P value***	DBP		***P*** value
	Noninvasive central (mmHg)	Sphygmomanometer (mmHg)		Noninvasive (mmHg)	Sphygmomanometer (mmHg)	
**Control (*n*** **= 10)**	**113.70 ± 7.84**	**123.00 ± 8.23**	0.001*	**80.70 ± 6.5**	**86.00 ± 6.7**	0.000*
**Gestational hypertension (*n*** **= 24)**	**129.95 ± 12.9**	**139.13 ± 16.49**	0.04*	**80.42 ± 8.6**	**89.58 ± 10.8**	0.01*
**Pre-existing hypertension (*n*** **= 7)**	**133.25 ± 13.2**	**135.28 ± 12.98**	0.04*	**84.86 ± 7.9**	**90.71 ± 5.34**	0.03*
**Preeclampsia (*n*** **= 69)**	**129.27 ± 15.45**	**140.37 ± 15.89**	0.000*	**83.10 ± 9.6**	**90.62 ± 9.1**	0.001*
**Total (*n*** **= 110)**	**126.39 ± 14.5**	**138.72 ± 16.64**	0.000*	**82.41 ± 9.0**	**89.00 ± 9.1**	0.001*

Data were expressed in the form of mean ± SD. *P* value was significant if < 0.05. *n* the number of patients, *DBP* diastolic blood pressure, *SBP* systolic blood pressure.* Significant values are labelled

### PWV and augmentation index

**Fig. 4:**
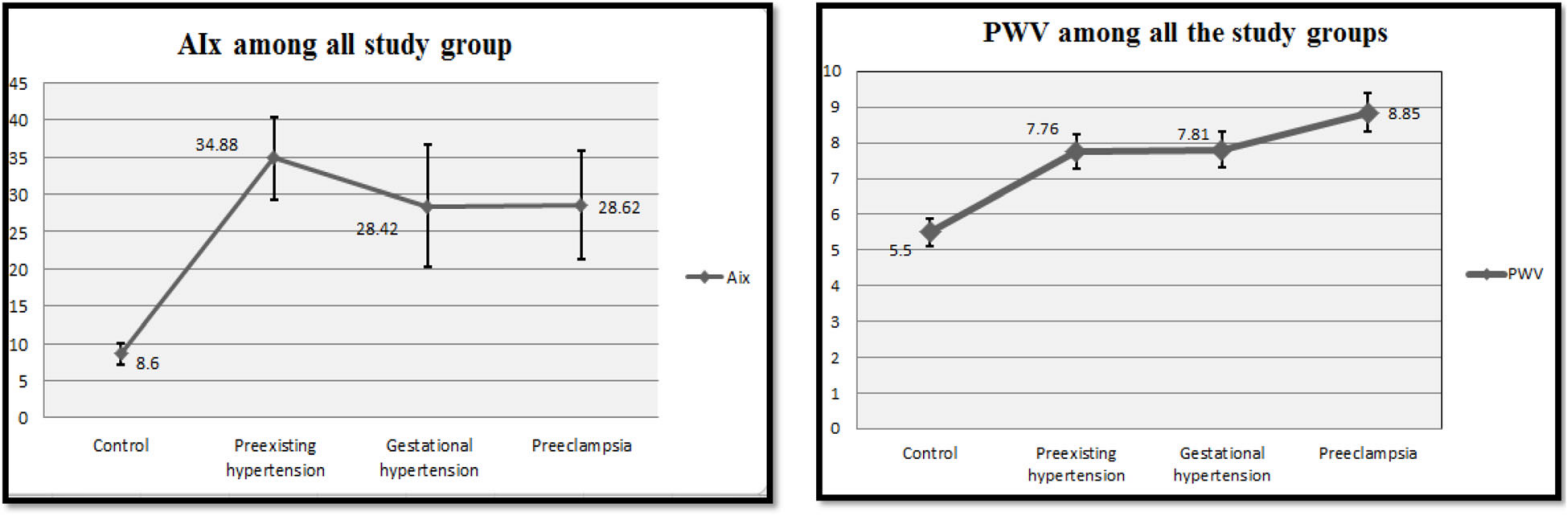
**a** Augmentation index in different study groups (ANOVA test of difference showed a significant *p* value < 0.001), and **b** pulse wave velocity in the different study groups (*p* = 0.03). AIx, augmentation index; PWV, pulse wave velocity

Both are considered indicators of arterial stiffness and were recorded from the non-invasive oscillometric device ( Mobil-O-Graph). Both parameters were compared between the three different hypertensive groups and the control group. AIx and PWV were significantly lower (*p* < 0.001 for AIx and 0.03 for PWV) in the control group, as shown in Fig. 4. Our reference for PWV normal value was 6.9 ± 1.8 and 23 ± 10.9 for AIx [13].

### Echocardiography-derived data

Illustrated in Table 1, it shows no significant difference regarding LA volume index, LV mass index, and LV EF by biplane Simpson’s. No significant differences were obtained in comparing different systolic echo measurements among the three different hypertensive groups. Patterns of LV geometry showed more frequent concentric remodeling in pre-eclampsia patients (44.9% of cases). Regarding LV diastolic dysfunction grades among the three studied hypertensive groups, normal diastolic function was found in 39 (39%) of all studied hypertensive patients. Grade 1 diastolic dysfunction was found in 58 (58%) of all patients, most found in the pre-eclampsia group 44 (76%). On the other hand, grade 2 diastolic dysfunction was found in only 3 (3%) patients with pre-eclampsia and gestational HTN. Patients with pre-eclampsia showed the lowest E/A ratio, septal and lateral e′ velocities, and the higher left atrial volume index.

### Complications and outcome of HDP

All 100 included patients had hospital delivery and were clinically followed up for pregnancy outcome and occurrence of complications. All patients completed their clinical follow-up. Most patients, 58 (58%), passed their pregnancy smoothly without complications. most complications were noticed in the pre-eclampsia group including (intrauterine growth retardation (IUGR) = eight patients, oligohydramnios = 4 patients, and HELLP syndrome = 13 patients). Eclampsia was found in 4 patients in the pre-existing hypertension group and 6 cases in the gestational hypertension group.

## Discussion

Little is known about the accuracy of non-invasive central blood pressure by an oscillometric automated device (Mobil-O-Graph) to the invasive blood pressure measurement by arterial cannulation conducted on the same population in hypertensive pregnant women.

Key findings of our study are as follows: (1) there was no difference between non-invasive BP readings by oscillometric automated devices (Mobil-O-Graph) and invasive BP readings, and (2) hypertension in pregnancy was associated with an increase in arterial stiffness, left ventricular diastolic dysfunction, and complications.

Regarding the main aim of the study, to assess the accuracy of non-invasive central BP measurements in comparison to invasive BP measurement, results showed no statistically significant difference between SBP and DBP measurements by the two methods between the different studied groups indicating that central blood pressure measured non-invasively by the oscillometric automated device (Mobil-O-Graph) is accurate as of the invasive assessment of blood pressure, and the strong positive correlation between non-invasive central blood pressure measurements and invasively measured both systolic BP and diastolic BP confirm this conclusion.

In agreement with our results, Gotzmann and colleagues [14] conducted a cross-sectional study of non-invasive central BP measurement by an oscillometric automated device (Mobil-O-Graph). It showed that the automated oscillometric monitors could assess central BP with acceptable accuracy. Their study was performed on 502 patients (228 women, 274 men) with a mean age of 67.9 ± 11.6 years undergoing elective coronary angiography. Their results revealed a highly significant positive correlation between invasively measured systolic (*r* = 0.763, *p* < 0.001) and diastolic (*r* = 0.618, *p* < 0.001) central blood pressures and non-invasive BP readings.

In the same context, another study, by Sanchez and colleagues [15], in 20 subjects (10 males (68 ± 12 years) and ten females (77 ± 8 years)), submitted for invasive coronary evaluations, showed a highly significant positive correlation between Mobil-O-Graph central BP, and the invasive BP values were found in men (*r* = 0.89) and women (*r* = 0.917).

Another study, by Weber and colleagues [16], included 30 patients undergoing elective coronary angiography for suspected coronary artery disease, mean age was 59 ± 11 years, non-invasive assessment of central SBP was performed by the same oscillometric automated device (Mobil-O-Graph) and invasive assessment during elective coronary angiography, and results revealed a high positive correlation to invasively measured systolic (*r=* 0.899, *p* < 0.001) central blood pressure in agreement with our results.

Of note, central hemodynamics recorded by the oscillometric device (Mobil-O-Graph) uses in the current study showed that AIx and PWV were higher in the pre-existing hypertension group with a significant difference when compared to non-hypertensive control.

Concordant to our results, Franz and colleagues [11] conducted a case-control study over 35 healthy pregnant women and 21 patients with pre-eclampsia; AIx and PWV were measured by an oscillometric device TensioClinic TL1 Arteriograph and TensioClinic software (TensioMed Ltd.) and found that the patients with pre-eclampsia had significantly elevated AIx values with *p* value 0.001, and the PWV values were higher in the preeclamptic groups but with a non-significant difference.

Furthermore, in Elvan-Tasšpinar and colleagues’ [17] study of 122 pregnant women divided into a normotensive group (51 women), hypertensive group (19 with chronic HTN, 19 with gestational HTN ), and preeclamptic group (31 women), PWV and AIx were measured non-invasively in all groups and results showed that the AIx and PWV were significantly higher in the hypertensive and preeclamptic group with *p* value < 0.05.

Despite being used routinely, sphygmomanometer BP has many limitations, as it does not represent real-life BP, in addition to the discrepancy between brachial BP and central BP with its predictive value for cardiovascular events [4]. Central BP was one of the explanations of the increased stroke risk in the atenolol arm in the landmark LIFE trial [5]. BP measurement via a catheter introduced into the artery (mostly radial or femoral artery) is called invasive or direct blood pressure measurement and is considered to be the most accurate method of blood pressure measurement [6].

In our study, we found a significant difference between sphygmomanometer and central methods in both SBP and DBP readings in patients with HDP, including pre-eclampsia. This was in concordance with Langenegger et al. who found discordance between readings collected by direct intra-arterial monitoring and peripheral methods by both manual and automated devices, and they concluded that invasive central BP monitoring is mandatory in patients with severe pre-eclampsia [18].

Regarding diastolic dysfunction, we found that diastolic dysfunction was more frequent in patients with HDP, including pre-eclampsia. In concordance with these results, Guirguis et al. found more frequent diastolic dysfunction in patients with pre-eclampsia. Furthermore, we demonstrated that other variants of HDP were also associated with diastolic dysfunction [19].

## Limitations

The invasive maneuver used (arterial cannulation) was refused by some patients, failed in other ones, and was an obstacle. We depended on radial cannulation (which is the standard in critical units) rather than advancing catheters into the aorta for accurate central BP assessment for comparison. The small number of non-pregnant, non-hypertensive matched the control due to logistic reasons. Lack of BP measurement, using the oscillometric automated device (Mobil-O-Graph), before and after BP control, could elucidate its role in an emergency. In this study, we did not evaluate the effect of different drugs used in HDP on BP control centrally or on pregnancy outcomes.

## Conclusion

Non-invasive measurement of central BP using oscillometric automated devices is as accurate as of the invasive methods, and it is a practical, safe method in pregnant women with hypertensive disorders. HDP was associated with an increase in arterial stiffness, left ventricular diastolic dysfunction, and complications.

## Data Availability

Data including excel sheets and BP results are available.

## Abbreviations

Aix: Augmentation index
ASE: American Society of Echocardiography
BP: Blood pressure
EAE: The European Association of Echocardiography
EF: Ejection fraction
HDP: Hypertensive disorders of pregnancy
HTN: Hypertension
LVH: Left ventricular hypertrophy
NI: Non-invasive
PWV: Pulse wave velocity
SBP: Systolic BP
